# Spin-polarized Second Harmonic Generation from the Antiferromagnetic CaCoSO Single Crystal

**DOI:** 10.1038/srep46415

**Published:** 2017-04-13

**Authors:** A. H. Reshak

**Affiliations:** 1 New Technologies - Research Centre, University of West Bohemia, Univerzitni 8, 306 14 Pilsen, Czech Republic; 2 School of Material Engineering, University Malaysia Perlis, 01007 Kangar, Perlis, Malaysia

## Abstract

The spin-polarized second harmonic generation (SHG) of the recently synthesized CaCoSO single crystal is performed based on the calculated electronic band structure. The calculation reveals that the spin-up (↑) channel of CaCoSO possesses a direct energy gap (Γ^v^-Γ^c^) of about 2.187 eV, 1.187 eV (K^v^-K^c^) for the spin-down (↓) channel and an indirect gap (Γ^v^-K^c^) of about 0.4 eV for the spin-polarized CaCoSO single crystal. The linear optical properties obtained reveal that the recently synthesized crystal exhibits considerable anisotropy with negative uniaxial anisotropy and birefringence favor to enhance the SHG. We have calculated the three non-zero tensor components of the SHG and found the 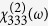 is the dominat component, one with a large SHG of about (*d*_33_ = 6.936 pm/V at *λ* = 1064 nm), the half value of KTiOPO_4_ (KTP). As the values of (↑)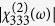 < (↓)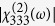 < spin-polarized 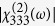 are related to the values of the energy gap of (↑) 2.187 eV> (↓) 1.187 eV> spin-polarized gap 0.4 eV; therefore, a smaller energy gap gives better SHG performance. Furthermore, the microscopic first hyperpolarizability, *β*_*ijk*_, is calculated.

A great effort has been made to synthesize novel nonlinear optical (NLO) crystals which are able to produce visible, UV and IR laser radiation at wavelengths that are presently inaccessible via conventional sources which have been in demand for several applications^1^,^2^,^3^,^4^. The NLO materials form a large group of semiconducting and dielectric materials with diverse optical, electrical, and structural properties. Some of these NLO materials appear to be promising candidates for optical frequency conversion applications in solid-state-based tunable laser systems^5^,^6^,^7^. These have potentially significant advantages over dye lasers because of their easier operation and the potential for more compact devices. Tunable frequency conversion in the mid-IR is based on optical parametric oscillators (OPO) using pump lasers in the near-IR. On the other hand, frequency doubling devices allow one to expand the range of powerful lasers in the far-IR. The transformation of frequencies within the wide spectral range of electromagnetic radiation (between 1–15 μm) for ultrafast lasers (from picoseconds up to femtoseconds) and large power densities can lead to significant progress in laser engineering. The method used for such frequency transformations is usually the generation of harmonic frequencies by employing high quality NLO crystals possessing large second optical susceptibilities^8^,^9^. In particular, the main method employed for frequency transformation is the second harmonic generation (SHG), where organic and inorganic crystals are now among the best^10^,^11^. Werake and Zhao^12^ have reported the observation of the SHG in GaAs induced by pure spin current. Takas and Aitken^13^ have presented the thermal analysis and SHG efficiency of α-Na_3_PO_3_S oxythiophosphate compound. Upon exposure to 1064 nm incident radiation, noncentrosymmetric α-Na3PO3S produces an SHG efficiency 200 times that of α-quartz and is nonphase-matchable (type 1). Wu *et al*.^14^ have synthesized acentric BaHgS_2_ by a conventional solid-state reaction method. It has been found that the powder of BaHgS_2_ exhibits a large SHG response of about 6.5 times compared with that of reference AgGaS_2_ at a fundamental wavelength (2.09 μm). Liu *et al*.^15^ have synthesized a new oxychalcogenide, BaGeOSe_2_ which contains acentric mixed-anion basic building units of GeO_2_Se_2_ tetrahedrons and BaOSe_6_ polyhedrons. These GeO_2_Se_2_ tetrahedrons and BaOSe_6_ polyhedrons are responsible for the large NLO response. Li *et al*.^16^ reported that the combination of the highly electropositive alkali metal (Na) and Zn with a *d*^10^ electronic configuration into a crystal structure affords one new IR NLO material, Na_2_ZnGe_2_S_6_, which exhibits excellent properties including a wide transparent region, a large band gap, and especially a balance between the strong NLO coefficient (30 × KDP) and high LDT (6 × AgGaS2), which indicate a promising application in the IR region. A few years ago it was reported that AgGaX_2_ (X = S, Se)^17^,^18^,^19^ and ZnGeP_2_^20^,^21^ exhibit good SHG coefficients for application in the IR region. In the UV and visible regions, great achievements have been made by providing many excellent NLO materials like *β*-BaB2O4 (BBO), LiB3O5 (LBO) and KBe2BO3F2 (KBBF)^22^,^23^,^24^,^25^,^26^. Kang *et al*.^27^ have designed two novel NLO carbonates KBeCO_3_F and RbAlCO_3_F_2_ using the first-principles theory. The investigated crystals are structurally stable and possesses very large energy band gaps, and considerable optical anisotropy. They reported that KBeCO_3_F and RbAlCO_3_F_2_ are very promising deep-UV NLO crystals alternative to KBBF. Liang *et al*.^28^ have investigated the usage of metal sulfides with diamond-like (DL) for NLO applications in the mid-IR spectral region. The linear and NLO properties of the DL-metal sulfides are analyzed on the basis of first-principles calculations. It has been found that it is relatively easy to achieve good balance between the band gap and the NLO performance. Moreover, moderate birefringence Δn (~0.03–0.10) is crucial for practical mid-IR NLO applications. They reported that several metal sulfides with normal DL and defect DL structures, show excellent mid-IR NLO properties. These provide an useful information for the design and discovery of novel materials possesses good mid-IR NLO performance. Lin *et al*.^29^ have reported that the *ab initio* approaches have the ability to accurately predict the optical properties in NLO crystals, and the developed analysis tools are very important to explore their intrinsic mechanism. This microscopic understanding is crucial for designing novel crystals with large NLO properties. It is expected that the first-principle approaches will deeply enhance the search efficiency and help the researchers to save resources in the exploration of novel NLO crystals with good performance.

Recently, the oxide chalcogenide compounds have become considerable candidates for several applications, for instance, as p-type transparent conductors^30^,^31^,^32^,^33^,^34^,^35^. Among these oxide chalcogenide compounds, the CaCoSO compound has been recently synthesized by Salter *et al*.^36^. It has been reported that the CaCoSO is isostructural with CaZnSO, BaCoSO and CaFeSO^36^,^37^,^38^,^39^,^40^. Since the newly synthesized CaCoSO crystallizes in non-centro-symmetry, this results in the loss of inversion symmetry, which in turn gives a considerable SHG. As the crystal structure of the CaCoSO single crystal has just been reported^36^ and there is no further information regarding its linear and nonlinear optical properties, we think it would be worthwhile to perform a comprehensive theoretical calculation to calculate the spin-polarized linear, and nonlinear optical properties and the microscopic first hyperpolarizability. Moreover, it is important to highlight that there is no previous report on the spin-polarized nonlinear optical properties and the microscopic first hyperpolarizability for the CaCoSO. In the current work, we aim to find new materials without inversion symmetry and possessing considerable SHG, which have been in demand for many industrial, medical, biological and entertainment applications.

## Density Functional Calculations

**Figure 1:**
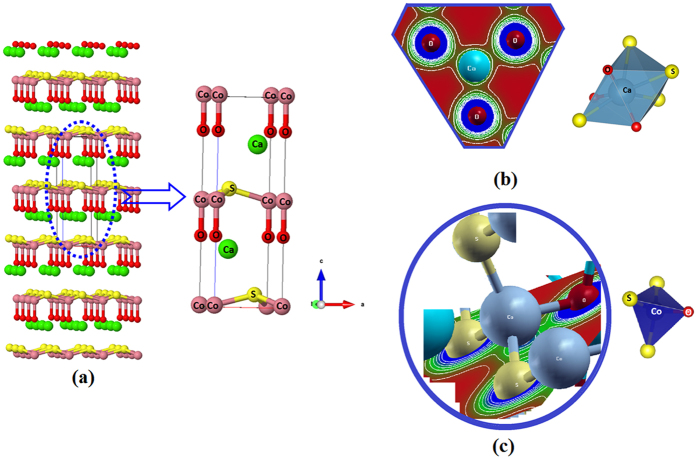
Crystal structure of the antiferromagnetic CaCoSO Single Crystal. (**b**) electron cloud in CaS_4_O_3_ (**c**) electron cloud in CoS_3_O tetrahedron Co-O strong covalent bond.

Very recently, Salter *et al*.^36^ have synthesized CaCoSO in the form of a single crystal with a *P6*_*3*_*mc* space group, cell parameters a = b = 3.7415(8) Å, c = 11.106(2) Å, V = 134.64(6) Å^3^ and Z = 2, at room temperature^36^. Utilizing the reported x-ray diffraction data of CaCoSO^36^, we performed comprehensive *ab-initio* calculations based on the full-potential method within the generalized gradient approximation (PBE-GGA) plus Hubbard Hamiltonian (U). In a step forward to gain accurate results, the experimental geometrical parameters^36^ are optimized using PBE-GGA^41^. The optimized crystal structure of CaCoSO single crystal is shown in Fig. 1(a–c). The resulting geometrical parameters are used to perform the calculations employing the *ab-initio* LAPW+ lo full-potential method utilizing the Wien2k code^42^,^43^,^44^. It is well known that for highly localized electrons, the Coulomb repulsion between the electrons in open shells should be taken into account; therefore, the Hubbard-like on-site repulsion should be added to the Kohn-Sham Hamiltonian. In the present work, we used the method of Anisimov *et al*.^45^ and Liechtenstein *et al*.^46^, where the Coulomb (U) and exchange (J) parameters are used. We applied the U on the 3*d* orbitals of the Co atom. Several U values are used to obtain a qualitative agreement with experimental data; we found 0.20 Ry is the optimal U value for Co-3*d*. In the current calculation, the self-consistency is obtained using 900 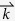 points in the irreducible Brillouin zone (IBZ). The self-consistent calculations are converged since the total energy of the system is stable within 0.00001 Ry. The spin-polarized linear and nonlinear optical properties and the microscopic first hyperpolarizability are calculated using 25000 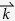 points in the IBZ, as accurate calculations require a dense sampling of the BZ.

## Results and Discussion

**Figure 2:**
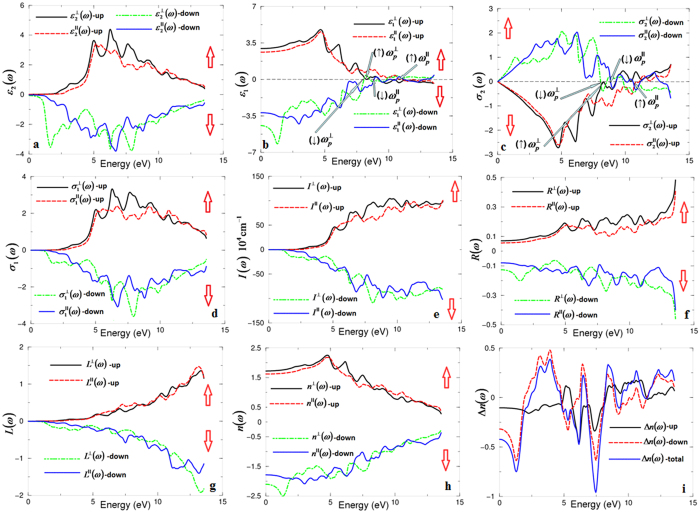
(**a**) Calculated (↑)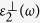 (dark solid curve-black color), (↑)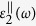 (light long dashed curve-red color), (↓)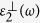 (light dotted dashed curve -green color) and (↓)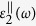 (dark solid curve-blue color); (**b**) Calculated (↑)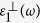 (dark solid curve-black color), (↑)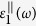 (light long dashed curve-red color), (↓)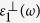 (light dotted dashed curve -green color) and (↓)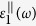 (dark solid curve-blue color); (**c**) Calculated (↑)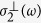 (dark solid curve-black color), (↑)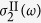 (light long dashed curve-red color), (↓)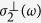 (light dotted dashed curve -green color) and (↓)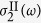 (dark solid curve-blue color); (**d**) Calculated (↑)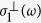 (dark solid curve-black color), (↑)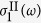 (light long dashed curve-red color), (↓)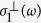 (light dotted dashed curve -green color) and (↓)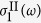 (dark solid curve-blue color); (**e**) Calculated (↑)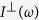 (dark solid curve-black color), (↑)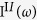 (light long dashed curve-red color), (↓)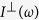 (light dotted dashed curve -green color) and (↓)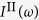 (dark solid curve-blue color); (**f**) Calculated (↑)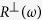 (dark solid curve-black color), (↑)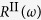 (light long dashed curve-red color), (↓)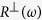 (light dotted dashed curve -green color) and (↓)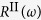 (dark solid curve-blue color); (**g**) Calculated (↑)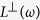 (dark solid curve-black color), (↑)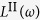 (light long dashed curve-red color), (↓)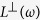 (light dotted dashed curve -green color) and (↓)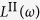 (dark solid curve-blue color); (**h**) Calculated (↑)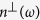 (dark solid curve-black color), (↑)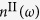 (light long dashed curve-red color), (↓)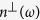 (light dotted dashed curve -green color) and (↓)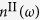 (dark solid curve-blue color); (**i**) Calculated (↑)Δ*n*(*ω*) (dark solid curve-black color), (↓)Δ*n*(*ω*) (light long dashed curve-red color) and total Δ*n*(*ω*) (dark solid curve-blue color).

The spin-polarized linear optical properties of the antiferromagnetic CaCoSO are calculated based on the spin-polarized electronic band structure. The complex dielectric function consists of real and imaginary parts^47^,^48^. For the hexagonal symmetry, the imaginary part consists of two tensor components: these are 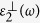 and 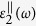. We have performed calculations for 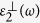 and 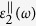 using the expression given elsewhere^47^,^48^. The calculated imaginary part for the spin-up (↑) and spin-down (↓) channels are shown in Fig. 2(a). Due to the fact that the spin-up channel has a different structure than that of the spin-down channel (see Fig. S1 Supplementary Materials), the resulting tensor components (↑)(↓)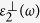 and (↑)(↓)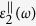 exhibit different spectral structures, following the allowed optical transitions between the occupied and unoccupied bands according to the selection rules. In order to identify the spectral structures of (↑)(↓)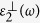 and (↑)(↓)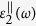, we need to look at the magnitude of the optical matrix elements for the spin-up and spin-down channels. The observed spectral structures in (↑)(↓)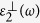 and (↑)(↓)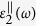 would correspond to those transitions that have large optical matrix elements. It has been noted that the fundamental absorption edge for (↑)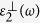 and (↑)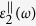 is situated at about 2.187 eV, whereas for (↓)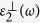 and (↓)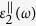 it is located at around 1.187 eV, which confirms that the spin-up channel possesses an energy band gap larger that that of the spin-down channel (see Fig. S1 Supplementary Materials). We should emphasize that the broadening is taken to be 0.1 eV, which is traditional for oxide crystals and is typical of the experimental accuracy. In the spin-up channel the fundamental absorption edge occurs due to the optical transition from Co-3p/3d, S-3p, and O-2p bands of the VBs to Ca-s/p, Co-4s/3p and O-2s bands of the CBs, while in the spin-down channel it occurs between Co-3p/3d, O-2p, S-3p VBs and Co-3p/3d, Ca-3p, O-2p, S-3p CBs. The main structure which is confined between 5.0 eV and 10.0 eV occurs due to the optical transitions between Ca-4s/3p, Co-4s/3p/3d, O-2s/2p, S-3s/3p VBs and Ca-4s/3p, Co-4s/3p, O-2s CBs in the spin-up channel. Whereas in the spin-down channel it is formed due to the transitions from Ca-4s/3p, Co-4s/3p, O-2s/2p, S-3s/3p VBs to Ca-4s/3p, Co-4s/3p/3d, O-2s CBs.

With the aid of the Kramers-Kronig relations^47^,^48^, the real parts of the optical dielectric function can be obtained from the imaginary part. Figure 2(b) illustrates the spectral features of (↑)(↓)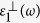 and (↑)(↓)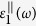. The calculated values of (↑)(↓)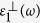 and (↑)(↓)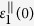 at the static limit are listed at Table 1, which shows that the values of (↓)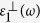 and (↓)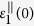 are grater than those of (↑)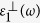 and (↑)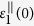. This confirms that the spin-down channel possesses an energy band gap smaller that that of the spin-up channel, following the Penn model 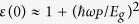 49, which shows that *ε*(0) is inversely proportional to the energy band gap. Furthermore, from the spectral structures of (↑)(↓)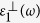 and (↑)(↓)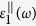, we can obtain the values of the uniaxial anisotropy (*δε*). These values are listed in Table 1 and show that the CaCoSO possesses negative *δε* for both channels. The plasma frequency can be obtained at the intersection points where (↑)(↓)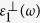 and (↑)(↓)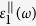 cross the energy axis (x-axis), as shown in Fig. 2(b). The calculated values of (↑)(↓)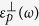 and (↑)(↓)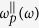 are listed in Table 1; these are the values of the plasmon maximum, which are the most intensive features in the optical spectrum.

From the calculated (↑)(↓)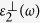, (↑)(↓)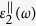, (↑)(↓)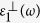 and (↑)(↓)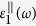, we can obtain (↑)(↓)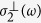, (↑)(↓)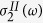, (↑)(↓)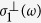 and (↑)(↓)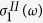, as shown in Fig. 2(c,d). The optical conductivity is directly related to the energy band structure of solids^50^; therefore, deep insight into the electronic structure of the materials can be further obtained from the optical conductivity. Furthermore, from the imaginary part of the optical conductivity, the (↑)(↓)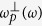 and (↑)(↓)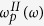 values can be obtained (Table 1).

Figure 2(e) illustrates the absorption coefficients of CaCoSO for the spin-up and spin-down channels. The fundamental absorption edge of the spin-up channel occurs at 2.187 eV, whereas it is 1.187 eV for the spin-down channel, which reveals that the absorption edge of the spin-down is smaller than that of the spin-up, confirming our previous observation at Fig. 2(a).

The reflectivity spectra (Fig. 2f) reveal that the CaCoSO possesses low reflectivity at the low energy range and the highest reflectivity at around 13.0 eV, which represents the lossless region. To support this statement the loss function is calculated and presented in Fig. 2(g). The refractive indices are very important quantities to determine the phase matching conditions which are necessary for the second harmonic generation. The spin-up/down refractive indices of the CaCoSO single crystal are calculated and presented in Fig. 2(h). From the parallel and perpendicular tensor components of the refractive indices, the birefringence can be obtained. The calculated spin-up/down and spin-polarized birefringence are presented in Fig. 2(i). The calculated values of the birefringence at the static limit and at the wavelength 1064 nm are listed at Table 1. It is clear that the CaCoSO single crystal exhibits relatively large birefringence. The electron clouds of the CaO_3_S_4_ and CoS_3_O (Fig. 1b,c) exhibit a planar shape with conjugated electron orbitals, which make the CoS_3_O groups the main source of the large birefringence in CaCoSO. It is well known that the birefringence determines partly whether an NLO material has the value of study^51^. It is clear that there is a considerable anisotropy between the two tensor components of the optical properties. The high electron density configuration and strong anisotropy for Co-S and Co-O indicate the main contribution of CoS_3_O to the optical anisotropy. The optical anisotropy is a favor to enhance the SHG.

**Figure 3:**
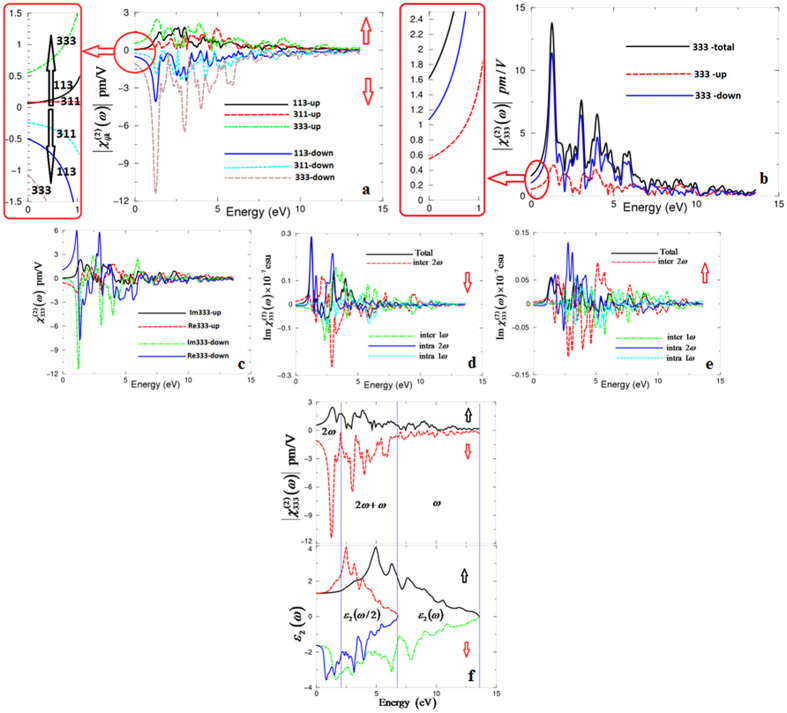
(**a**) Calculated (↑)(↓)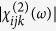 for the three tensor components of CaCoSO single crystal; (**b**) Calculated (↑)(↓)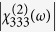 and the total 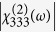 the dominate tensor component of CaCoSO single crystal; (**c**) Calculated (↑)(↓) of the imaginary and real parts of 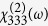 the dominate tensor component of CaCoSO single crystal; (**d**) Calculated (↑) of the imaginary part of 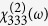 spectrum (dark solid curve-black color) along with the intra (2*ω*)/(1*ω*) (light solid curve-blue color)/(light dashed doted curve-cyan color) and inter (2*ω*)/(1*ω*) (light long dashed curve-red color)/(light doted curve-green color) -band contributions, here all Im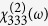 are multiplied by 10^−7^, in esu units; (**e**) Calculated (↓) of the imaginary part of 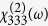 spectrum (dark solid curve-black color) along with the intra (2*ω*)/(1*ω*) (light solid curve-blue color)/(light dashed doted curve-cyan color) and inter (2*ω*)/(1*ω*) (light long dashed curve-red color)/(light doted curve-green color) -band contributions, here all Im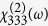 are multiplied by 10^−7^, in esu units; (**f**) ***–**upper panel-* Calculated (↑)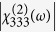 (dark solid curve-black color) and (↓)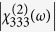 (light long dashed curve-red color); ***-**lower panel-* Calculated (↑)*ε*_2_(*ω*) (dark solid curve-black color); Calculated (↑) *ε*_2_(*ω*/2) (dark dashed curve-red color); Calculated (↓)*ε*_2_(*ω*/2) (light dotted dashed curve -green color); Calculated (↓)*ε*_2_(*ω*/2) (dark solid curve-blue color).

Furthermore, we have calculated the spin-up (↑) and spin-down (↓) and the spin-polarized second harmonic generation for the CaCoSO single crystal. The formalisms which are used to calculate the complex nonlinear optical are presented elsewhere^52^,^53^,^54^. The investigated single crystal possesses three non-zero tensor components. These are 113, 311 and 333; only one of these tensor components is the most intensive one and acts as the dominant one. From the calculated (↑)(↓)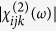 (Fig. 3a), we can notice that the (↑)(↓)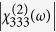 is the most intensive among the others; therefore, 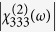 is the dominant due to the crystal’s symmetry. Figure 3(b) illustrates the dominant tensor component for the spin-up, spin-down and the spin-polarized 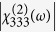 for the CaCoSO single crystal. The SHG values of the three tensor components are listed in Table 2, which shows that the investigated crystal possesses a large SHG of about 1.628 pm/V at the static limit and 13.872 pm/V (*d*_33_ = 6.936 pm/V) at *λ* = 1064 nm. The obtained value of the SHG at *λ* = 1064 nm is about the half value of the well-known NLO crystal KTiOPO_4_ (KTP)^55^. Following Table 2, the (↑)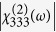 < (↓)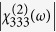 < spin-polarized 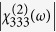 is related to the value of the energy gap (↑) 2.187 eV> (↓) 1.187 eV> spin-polarized gap 0.4 eV; therefore, we can see that our finding is supported by the statement: a smaller energy gap gives better SHG performance.

The dominant tensor component 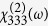 consists of imaginary and real parts as shown in Fig. 3(c) for the spin-up, spin-down channels. The imaginary and real parts further consist of 2*ω*/*ω* inter-/intra-band contributions as shown in Fig. 3(d,e). In order to understand the origin of the spectral features of the dominant tensor component 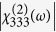, the imaginary part of the dielectric function as a function of *ω*/2 and *ω* is associated with 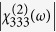, as shown in Fig. 3(f). This clearly shows that the structure below the half energy gap (1.093 eV(↑), 0.593 eV(↓)) is associated with 2*ω* resonance, whereas the structure above the fundamental energy gap (2.187 eV(↑), 1.187 eV(↓)) is associated with interference between 2*ω* and *ω* resonances and the tail is mainly due to *ω* resonance. The values of the microscopic first hyperpolarizability, *β*_*ijk*_^56^,^57^, vector component along the dipole moment direction for the dominate tensor component are calculated at the static limit and at *λ* = 1064 nm, as shown in Table 2.

## Conclusions

Based on the experimental crystallographic data of the recently synthesized CaCoSO single crystal, the spin-polarized SHG is calculated and the origin of the large SHG is discussed in detail. It has been found that the spin-up channel of the CaCoSO single crystal possesses direct energy (Γ^v^-Γ^c^) of about 2.187 eV, 1.187 eV (K^v^-K^c^) for the spin-down channel and an indirect gap (Γ^v^-K^c^) of about 0.4 eV for the spin-polarized CaCoSO single crystal. The linear optical properties reveal that the recently synthesized crystal exhibits considerable anisotropy with negative uniaxial anisotropy and birefringence favor to enhance the SHG. It is clear that CaCoSO single crystal exhibits relatively large birefringence. The electron clouds of the CaO_3_S_4_ and CoS_3_O groups exhibit a planar shape with conjugated electron orbitals, which make the CaO_3_S_4_ and CoS_3_O groups the main source of the large birefringence in CaCoSO. It is well known that the birefringence determines partly whether an NLO material has the value of study. The high electron density configuration and strong anisotropy for Co-S and Co-O indicate the main contribution of CaO_3_S_4_ and CoS_3_O to the optical anisotropy. The optical anisotropy is a favor to enhance the SHG. We have calculated the three non-zero tensor components of the SHG and found the 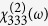 is the dominant one, with a large SHG of about (*d*_33_ = 6.936 pm/V at *λ* = 1064 nm), the half value of the well-known NLO crystal KTiOPO_4_ (KTP). The (↑)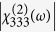 < (↓)
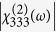
< spin-polarized 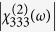 is related to the value of the energy gap of the (↑) 2.187 eV> (↓) 1.187 eV> spin-polarized gap 0.4 eV; therefore, we can see that our finding is supported by the statement: a smaller energy gap gives better SHG performance. Furthermore, the values of the microscopic first hyperpolarizability, *β*_*ijk*_, for the dominant tensor component is calculated at the static limit and at *λ* = 1064 nm.

## Additional Information

**How to cite this article**: Reshak, A. H. Spin-polarized Second Harmonic Generation from the Antiferromagnetic CaCoSO Single Crystal. *Sci. Rep.* **7**, 46415; doi: 10.1038/srep46415 (2017).

**Publisher's note:** Springer Nature remains neutral with regard to jurisdictional claims in published maps and institutional affiliations.

## Supplementary Material

Supplementary Materials

## Figures and Tables

**Table 1:** The calculated energy band gap, 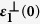, 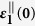, *δε*, 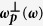, 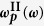, 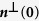, 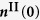 and Δ*n*(0).

	CaCoSO	
	Spin-up	Spin-down
Eg (eV)	2.187	1.187
Spin-polarized Eg (eV)	0.4	
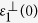	2.968	4.420
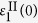	2.621	3.184
*δε*	−0.124	−0.325
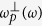	9.292	8.068
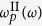	11.115	10.054
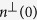	1.722	2.102
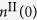	1.619	1.784
Δ*n*(0)	−0.103 (Δ*n*(*ω*) = −0.125 at *λ* = 1064 nm)	−0.318 (Δ*n*(*ω*) = −0.595 at *λ* = 1064 nm)
Δ*n*(0) total	−0.422 (Δ*n*(*ω*) = −0.708 at *λ* = 1064 nm)	

**Table 2:** Calculated spin-polarized 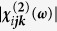 and *β*_*ijk*_ of CaCoSO, in pm/V at static limit and at *λ* = 1064 nm.

	Spin-up		Spin-dn		Spin-up at *λ* = 1064 nm		Spin-dn at *λ* = 1064 nm	
	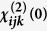	*d*_*ijk*_ = 0.5 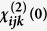	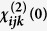	*d*_*ijk*_ = 0.5 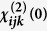	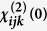	*d*_*ijk*_ = 0.5 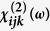	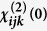	*d*_*ijk*_ = 0.5 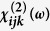
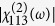	0.064	*d*_*15*_ = 0.032	0.499	*d*_*15*_ = 0.249	0.783	*d*_*15*_ = 0.391	3.656	*d*_*15*_ = 1.828
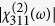	0.079	*d*_*31*_ = 0.039	0.240	*d*_*31*_ = 0.120	0.126	*d*_*31*_ = 0.063	1.270	*d*_*31*_ = 0.635
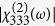	0.549	*d*_*33*_ = 0.275	1.071	*d*_*33*_ = 0.535	2.167	*d*_*33*_ = 1.083	10.433	*d*_*33*_ = 5.216
Spin-polarized 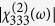	1.628 (*d*_*33*_ = 0.814)				13.872 (*d*_*33*_ = 6.936)			
*β*_333_	0.0803 × 10^−30^ esu		0.1563 × 10^−30^ esu		0.3225 × 10^−30^ esu		1.5867 × 10^−30^ esu	
Spin-polarized *β*_333_	0.2317 × 10^−30^ esu				1.9215 × 10^−30^ esu			

Where 1 pm/V = 2.387 × 10^−9^ esu.
