# Supplementary material for: Spin-polarized Second Harmonic Generation from the Antiferromagnetic CaCoSO Single Crystal
Source: Sci Rep. 2017 Apr 13;7:46415. doi: 10.1038/srep46415 (PMC5390297; doi:10.1038/srep46415)
Supplement: Supplementary Materials [file srep46415-s1.doc]

**Spin-polarized Second Harmonic Generation from the Antiferromagnet CaCoSO Single Crystal**

A. H. Reshak ^1,2,*^

^1^New Technologies - Research Centre, University of West Bohemia, Univerzitni 8, 306 14 Pilsen, Czech Republic

^2^ School of Material Engineering, University Malaysia Perlis, 01007 Kangar, Perlis, Malaysia

**
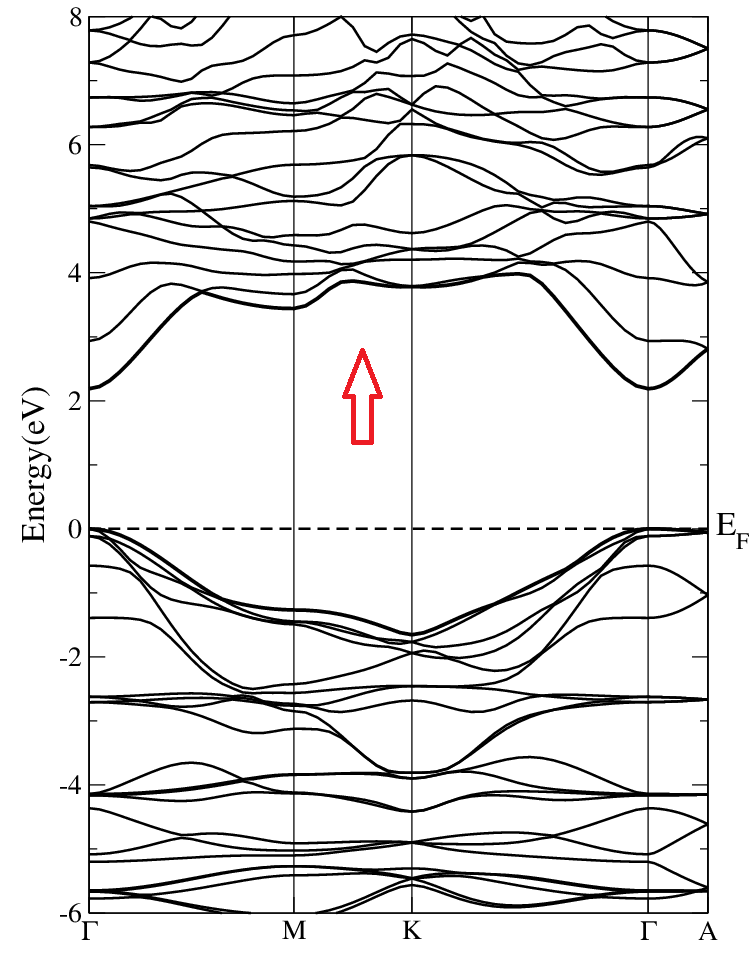

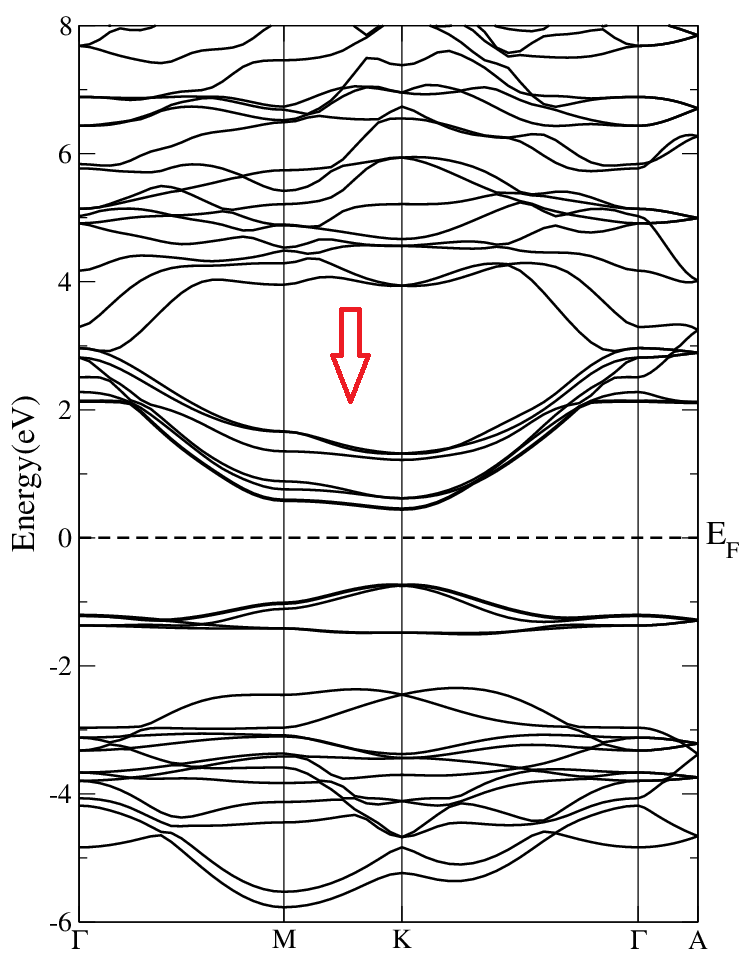
**

**(a) (b)**

**
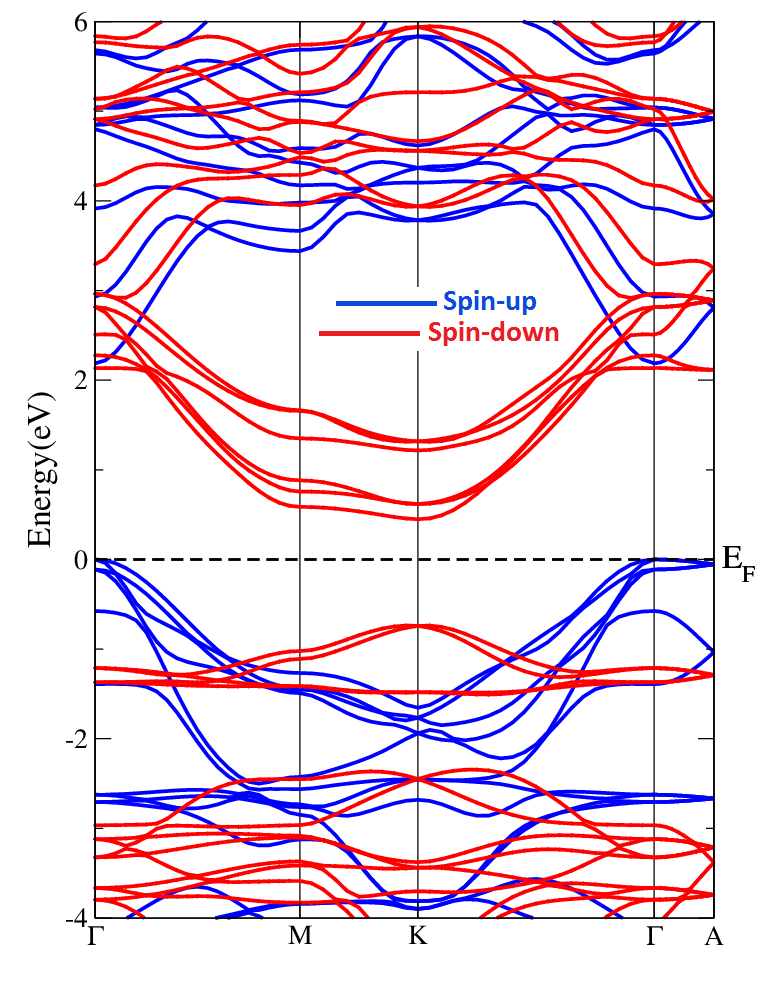
**

**(c)**

**Fig. S1:** Spin-polarized electronic band structure of antiferromagnet CaCoSO single crystal; (a) Spin-up; (b) Spin-down; (C) Spin-polarized electronic band structure.
